# *PLAGL1* is associated with prognosis and cell proliferation in pancreatic adenocarcinoma

**DOI:** 10.1186/s12876-022-02609-y

**Published:** 2023-01-04

**Authors:** Xing Liang, Zhiping Fu, Liang Tang, Minghui Zheng, Danlei Chen, Anan Liu, Ligang Shi, Linhua Yang, Chenghao Shao, Xiaoqiang Dong

**Affiliations:** 1 Department of General Surgery, The First Affiliated Hospital of Soochow University, Suzhou, 215006 Jiangsu Province China; 2 Department of Pancreatic-Biliary Surgery, Second Affiliated Hospital of Naval Medical University, Fengyang Road 415, Shanghai, 200003 China; 3 Department of Biliary-Pancreatic Surgery, Renji Hospital, School of Medicine, Shanghai Jiao Tong University, Shanghai, 200127 China

**Keywords:** Pancreatic adenocarcinoma, *PLAGL1*, Cell proliferation, Prognostic biomarker, Overall survival

## Abstract

**Background:**

Emerging evidence has shown the crucial roles of *pleomorphic adenoma gene* (*PLAG*) family genes in multiple cancers. However, their functions and mechanisms in pancreatic adenocarcinoma (PAAD) remain poorly understood.

**Methods:**

We analyzed the expression levels of *PLAG* family genes in both The Cancer Genome Atlas (TCGA) database and a Gene Expression Omnibus (GEO) database, and confirmed the results in our three independent cohorts of 382 PAAD tissues and 362 adjacent nontumor pancreatic tissues. Integrated analyses were carried out to explore the function, mechanism and prognostic value of the selected *PLAG* family gene in PAAD patients.

**Results:**

By analyzing the TCGA and GEO databases, *PLAGL1* was identified to be downregulated in PAAD tissues, and its decreasing levels of both mRNA and protein were verified in our three independent PAAD cohorts. PLAGL1 expression was inversely correlated with clinicopathological factors including the Ki67^+^ cell rate and pathologic stage. Further GSEA of the TCGA-PAAD cohort demonstrated that multiple signaling pathways implicated in cell proliferation were enriched in the lower *PLAGL1* expressing PAAD group. Moreover, we demonstrated that *PLAGL1* expression was obviously negatively associated with patients’ overall survival outcome in both the TCGA-PAAD cohort and our verification cohorts. Additionally, through MTS and BrdU assays, we further demonstrated in vitro that PLAGL1 had the impact of preventing the proliferation of pancreatic cancer cells.

**Conclusions:**

Our present study suggested that downregulated *PLAGL1* might act as a biomarker in predicts poor prognosis and one of important factors in increasing cell proliferation in PAAD. This study provides us with a novel prognostic marker and therapeutic strategy for PAAD, which deserves further study.

**Supplementary Information:**

The online version contains supplementary material available at 10.1186/s12876-022-02609-y.

## Introduction

Pancreatic adenocarcinoma (PAAD) is one of the leading fatal digestive malignancies in the world. It ranks the fourth in the lethality of common neoplasms [1] and has a 5-year survival rate less than 10% [2], and therapeutic efficacy and overall survival (OS) have undergone little clear improvement in the last two decades. Therefore, the need to determine the mechanisms of PAAD malignant phenotypes and to identify novel effective prognostic and therapeutic targets is urgent.

As a member of a subfamily of *zinc finger proteins* (*ZFPs*), the *pleomorphic adenoma gene* (*PLAG*) family has three members: *PLAG1*, *PLAG-like 1* (*PLAGL1*) and *PLAG-like 2* (*PLAGL2*) [3]. Many studies have confirmed that all three genes in the *PLAG* family play critical roles in regulating the expression of tumor-related genes as transcription factors of nuclear proteins or cofactors of other proteins. With high structural homology, slightly different DNA-binding specificities of the three *PLAG* family proteins result in their different functions. The binding capacities of PLAG1 and PLAGL2 are difficult to distinguish, and the consensus binding sequence has been confirmed to be GRGGC(N)6-8RGGK [4], while the consensus binding sequence of PLAGL1 was GGGGGGCCCC [5], lacking the G-cluster downstream binding sequence of *PLAG1* and *PLAGL2*. As a result, the function of *PLAGL1* is slightly different from that of *PLAG1* and *PLAGL2*. As an imprinting gene, while *PLAG1* and *PLAGL2* are not*, PLAGL1* has been detected to be widely expressed in human organs from embryo to adult [5] and was confirmed as a tumor suppressor gene in multiple cancers, such as large B-cell lymphoma, lung, gastric, colorectal, breast, ovarian and prostate cancer [6–10]. Its main mechanism might be regulating cell cycle progression and cell differentiation via the *p53* and *p21* pathways [11, 12]. However, recent studies have provided evidence suggesting that *PLAGL1* might also play a tumorigenic function, such as in glioblastoma and clear cell renal cell carcinoma, and others [13–15]. Thus, the functions and mechanisms of *PLAGL1* in different tumors are still unclear and deserve further exploration. Needless to say, it is scarcely reported in PAAD.

In this study, we detected the expression of *PLAG* family genes in The Cancer Genome Atlas (TCGA) and Gene Expression Omnibus (GEO) datasets, verifying the significantly decreased level of *PLAGL1* in PAAD tissues, compared with normal pancreatic tissues. Our three independent PAAD cohorts confirmed this result. Clinicopathological analysis revealed that the PLAGL1 protein expression level was correlated with Ki67^+^ expression and pathologic stage in PAAD. Gene set enrichment analysis (GSEA) of the TCGA-PAAD cohort also indicated that decreased *PLAGL1* might be one of important factors in increasing cell proliferation in PAAD. Furthermore, it was proven to be of prognostic value for predicting PAAD patients’ overall survival in both the TCGA-PAAD cohort and our verification cohorts. Finally, we found a decreased proliferation rate of pancreatic cancer cells with PLAGL1 overexpression in vitro. Consequently, these findings may contribute to the discovery of a new prognostic marker and a potential therapeutic target related to *PLAGL1* for patients suffering from PAAD.

## Materials and methods

### Tissue samples

In total, 382 PAAD tissue samples and 362 adjacent nontumor pancreatic tissue samples were collected in this study. Fifty-eight pairs of fresh PAAD and adjacent nontumor pancreatic tissues (cohort 1) and 224 pairs of formalin-fixed paraffin-embedded (FFPE) PAAD and adjacent nontumor pancreatic tissues (cohort 3) were collected from the Department of Biliary-Pancreatic Surgery at Renji Hospital; 100 FFPE PAAD tissues and 80 FFPE adjacent nontumor pancreatic tissues were obtained from Shanghai OutdoBiotech Ltd. (cohort 2). None of the patients in this study had received radiotherapy, chemotherapy or other antitumor therapies before surgery. Pathological diagnoses were confirmed by two certified pathologists. For Cohort 1, there were no follow-up data. For Cohort 2, there were no patients who were lost to follow-up. There were 238 patients in all when Cohort 3 cases were being collected, and 14 patients were lost to follow-up. 5.9% (14/238) of cases were lost to follow-up. Overall survival (OS) was defined as the postoperative time until the patient’s death or last follow-up. Disease-free survival (DFS) was defined as the postoperative time until the recurrent tumor was detected by radiography. The clinical characteristics of 382 PAAD patients in cohorts 1 to 3 are listed in Additional file 1: Table S1, as we previously reported [16].

### Quantitative polymerase chain reaction (qPCR)

Total RNA of PAAD and adjacent nontumor pancreatic tissue samples was prepared using TRIzol reagent (AM9738, Sigma-Aldrich, MO, USA) according to the manufacturer’s protocol as follows. Tissue samples was grinded with liquid nitrogen, and was homogenized with 1 ml of TRIzol reagent (AM9738, Sigma-Aldrich, MO, USA) per 50–100 mg of tissue. Homogenized samples were incubated for 5 min at room temperature. Insoluble material was removed by centrifugation at 12,000 ×*g* for 10 min at 2 to 8 °C. Add 0.2 ml of chloroform per 1 ml of TRIzol reagent into the supernatant. Shake for 15 s and incubate them at room temperature for 2 to 3 min. Centrifuge the samples at no more than 12,000 ×*g* for 15 min at 2 to 8 °C, and transfer the aqueous phase to a fresh tube. Add 0.5 ml of isopropyl alcohol per 1 ml of TRIzol reagent and incubate at room temperature for 10 min. After centrifuging at no more than 12,000 ×*g* for 10 min at 2 to 8 °C, the RNA precipitate was obtained. Wash and redissolve the RNA pellet, preparing for use next. Then, cDNA was reverse transcribed based on 1 μg RNA using the PrimerScript RT Reagent Kit (RR037A, Takara Bio, Beijing, China). SYBR Premix Ex Taq (RR420A, Takara Bio) was used for qPCR. Relative mRNA expression levels in clinical samples were calculated by 2^−△△Ct^ and normalized to *GAPDH*. All the primers are presented in Additional file 2: Table S2.

### Immunohistochemical (IHC) staining of tissue microarrays

The method of IHC staining was the same as that in our previous research [16]. Briefly, 5-μm-thick paraffin sections of tissue microarrays were prepared. After deparaffinization with xylene and graded alcohols, the sections were treated with 3% H_2_O_2_ and heat-induced with 10 mM citric sodium (pH 6.0) to retrieve antigens. Then, 3% BSA was used to cover the objective tissues at room temperature for 30 min. Moving away the blocking solution, the sections were incubated with primary antibodies at 4 ℃ overnight, followed by secondary antibodies at room temperature for one hour. 3,3-diaminobenzidine tetrahydrochloride was used as colouring reagent, and haematoxylin was used as a counterstain for nuclei. An Microscopy (CIC, XSP-C204) and an Olympus camera was used to photograph the stained slides. The primary antibodies used in the study were as follows: PLAGL1 (SANTA CRUZ, sc-166944, 1:50) and Ki67 (Sigma, ZRB1007, 1:200). The staining intensity of PLAGL1 was quantified (I score: negative, 0; weakly positive, 1; moderately positive, 2; strongly positive, 3), the same as the staining quantity of PLAGL1 (Q score: 0–5% positively stained cells, 0; 6–35%, 1; 36–70%, 2; and > 70%, 3). Then, the final score (S score) of PLAGL1 staining was calculated by the I score × Q score. Cases with an S score ≥ 4 were divided into a high PLAGL1 expression group, and those with as S score < 4 were divided into a low PLAGL1 expression group. The staining intensity and quantity of PLAGL1 were scored by two certified pathologists independently in a blinded manner.

### Bioinformatic analysis

The gene expression data and survival data of PAAD patients in TCGA database were available on the internet (https://portal.gdc.cancer.gov/). GSE28735 was downloaded from the GEO (https://www.ncbi.nlm.nih.gov/geo/) database. Gene set enrichment analysis (GSEA, https://www.gsea-msigdb.org/gsea/index.jsp) was used to predict the significant gene sets and neighborhoods of *PLAGL1* in the TCGA-PAAD cohort. Normalized enrichment scores (NES) > 1 and *P* < 0.05 were considered to be significant.

## Cell culture

Human pancreatic cancer cell lines (PANC-1 and BXPC-3) were purchased from Cell Resource Centre of Shanghai Institutes for Biological Sciences, Chinese Academy of Sciences (Shanghai, China), and cultured in RPMI-1640 (21870076, Gibco, NY, USA), which was supplemented with 10% fatal bovine serum in a humidified atmosphere of 5% CO2 at 37 °C. The duration between cell line freezing and experiment use was no more than 4 weeks.

### Cell transfection and proliferation assays

*PLAGL1* overexpression and vector control plasmid were purchased from MiaolingBio (Wuhan, China), and were transfected into pancreatic cancer cells using Lipofectamine 2000 (Invitrogen, USA) following the manufacturer's instruction. After transfecting, pancreatic cancer cells were seeded in 96-well plates (1000 cells per well) and cultured at 37 °C for 96 h. Then the MTS solution (G3581, Prom- ega, WI, USA) was added to each well of the plate at the different time points, followed by incubating at 37 °C for 1 h. The absorbance at 490 nm was measured in a Synergy 2 microplate reader (Biotek, VT, USA).

### Bromodeoxyuridine (BrdU) assay

The transfected (for 72 h) PANC-1 and BXPC-3 cells were incubated with 10 µmol/l BrdU (B9258, Sigma) for 4 h at 37 °C. The cells were then stained with BrdU Mouse mAb (product no. 5292; 1:200; Cell Signaling Technology, Inc.) at 4 °C overnight and goat anti-mouse IgG Alexa Flour 488 conjugated (ab150113; 1:500; Abcam) at room temperature for 1 h. DAPI (D9542, Sigma) was used to stain the nucleus. Images were obtained using Lecia DM2500 fluorescence microscope.

### Statistical analysis

Statistical analysis was calculated by SPSS 17.0 software (IBM, IL, USA). Data in this research are shown as the mean ± standard deviation (SD). For analyzing normally distributed quantitative variables, Student’s t test was performed. Chi-square or Fisher’s exact tests were performed as appropriate for the analysis of categorical variables. Multivariable logistic regression was carried out to detect the association of PAAD clinicopathological factors with PLAGL1 expression. To compare the two survival curves of the *PLAGL1* high-expression and low-expression groups, the log-rank test was performed. A Cox proportional hazards regression model was applied to analyze the independent prognostic factors of PAAD. Hazard ratios (HRs), 95% confidence intervals (CIs) and *P* -values were calculated. If *P* < 0.05, the result was considered significant. (*P < 0.05; **P < 0.01; ***P < 0.001).

## Results

### Analysis of *PLAG* family differential expression in PAAD patients

**Fig. 1:**
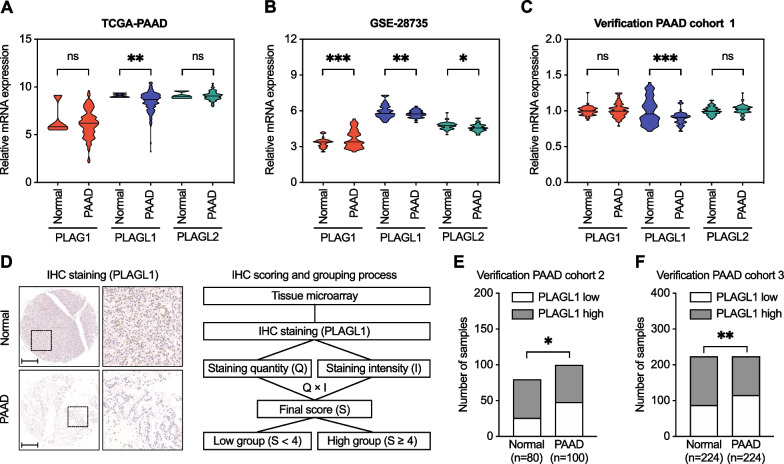
Expression levels of *PLAG* family genes in PAAD. **a**–**c** The expression levels of *PLAG* family genes in PAAD tissues and normal pancreatic tissues in the TCGA-PAAD cohort, GEO (GSE28735) cohort, and our verification PAAD cohort 1. **d** Left: Representative IHC staining of PLAGL1 protein high/low expression in PAAD and adjacent nontumor pancreatic tissues; Right: the progress of PLAGL1 IHC scoring evaluation and grouping process. **e**–**f** Histogram of the number of samples classied by the PLAGL1 IHC final score of PAAD and adjacent nontumor pancreatic tissues in our PAAD verification cohort 2 and cohort 3. Scale bar = 200 μm; ***P < 0.001; **P < 0.01; *P < 0.05; ns, not significant; Student’s t test

To explore the potential roles of the *PLAG* family genes in PAAD malignant phenotypes, we first analyzed the aberrant expression levels of the three *PLAG* family genes based on TCGA database and a GEO database (GSE28735) (Fig. 1a, b). Compared with normal tissues of the pancreas, only the *PLAGL1* expression level in PAAD tissues was significantly reduced in both the TCGA and GEO cohorts (*P* < 0.01). The expression level of *PLAGL2* was also significantly reduced in the GSE28735 GEO database (*P* < 0.05), while *PLAG1* was significantly increased (*P* < 0.001). However, the expression levels of *PLAG1* and *PLAGL2* showed no significant difference between PAAD and normal pancreas samples in the TCGA cohort.

To confirm the expression levels of these three genes in the TCGA and GEO cohorts, we next detected their mRNA levels in our 58 cases of fresh PAAD and paired tumor-adjacent samples by qPCR (cohort 1). Only *PLAGL1* was still significantly reduced in PAAD tissues (*P* < 0.001) (Fig. 1c). *PLAG1* and *PLAGL2* did not achieve the same significance level as the TCGA cohort. We also performed tissue microarray IHC staining of PLAGL1 in two other independent cohorts of PAAD and pancreatic tumor-adjacent tissues. The results indicated that the PLAGL1 protein level in PAAD tissues was decreased in cohorts 2 and 3 (*P* < 0.05 and *P* < 0.01, respectively) (Fig. 1d–f). Consequently, we suggested that *PLAGL1* was statistically significantly downregulated in PAAD.

### Correlations between *PLAGL1* expression and clinicopathological features in PAAD patients

**Fig. 2:**
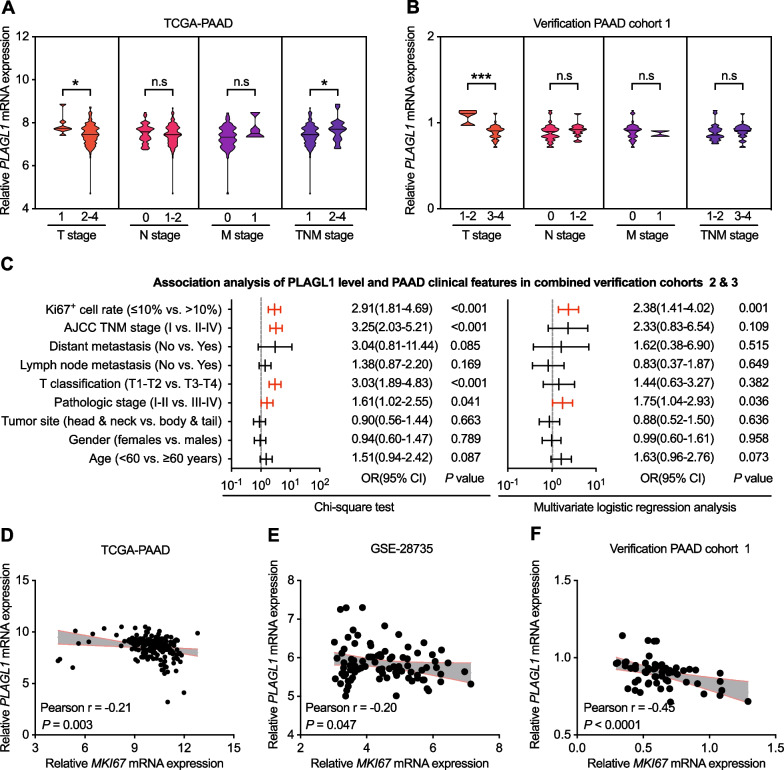
The correlation between PLAGL1 expression and PAAD patients’ clinicopathological factors. **a**–**b** The correlation between *PLAGL1* mRNA expression and TNM stages in the TCGA-PAAD cohort and our PAAD verification cohort 1. **c **Chi-square test and multivariate logistic regression model for predicting the correlation between PLAGL1 expression and clinicopathological factors of our combined PAAD cohorts 2 and 3. **d**–**f** Relationship between *PLAGL1* and *MKI67* expression levels in the TCGA-PAAD cohort, GEO (GSE28735) cohort, and our PAAD verification cohort 1. ***P < 0.001; *P < 0.05; ns, not significant; Student’s t test

As *PLAGL1* expression was decreased in PAAD tissues as confirmed above, we wondered whether the level of *PLAGL1* was related to PAAD patients’ clinicopathological factors. As shown in Fig. 2a, b, the *PLAGL1* expression level was negatively correlated with T stage in both the TCGA-PAAD cohort and our PAAD verification cohort 1, but not correlated with N/M stage in the two cohorts. Moreover, the chi-square test and multivariate logistic regression analysis performed in our other two combined PAAD verification cohorts 2 and 3 revealed that a reduced protein level of PLAGL1 was correlated with a high Ki67^+^ cell rate and pathologic stage (Fig. 2c, Additional file 3: Table S3, Additional file 4: Table S4, Additional file 5: Table S5). The relationship between *PLAGL1* and *MKI67* expression levels also confirmed that the mRNA expression level of *PLAGL1* was negatively correlated with *MKI67* mRNA expression levels in the TCGA-PAAD cohort, GSE28735 cohort and our PAAD verification cohort 1 (Fig. 2d–f). Taken together, the results suggested that *PLAGL1* might work as a tumor suppressor gene regulating tumor growth and cell proliferation in PAAD.

### Functional analysis of *PLAGL1* in PAAD

**Fig. 3:**
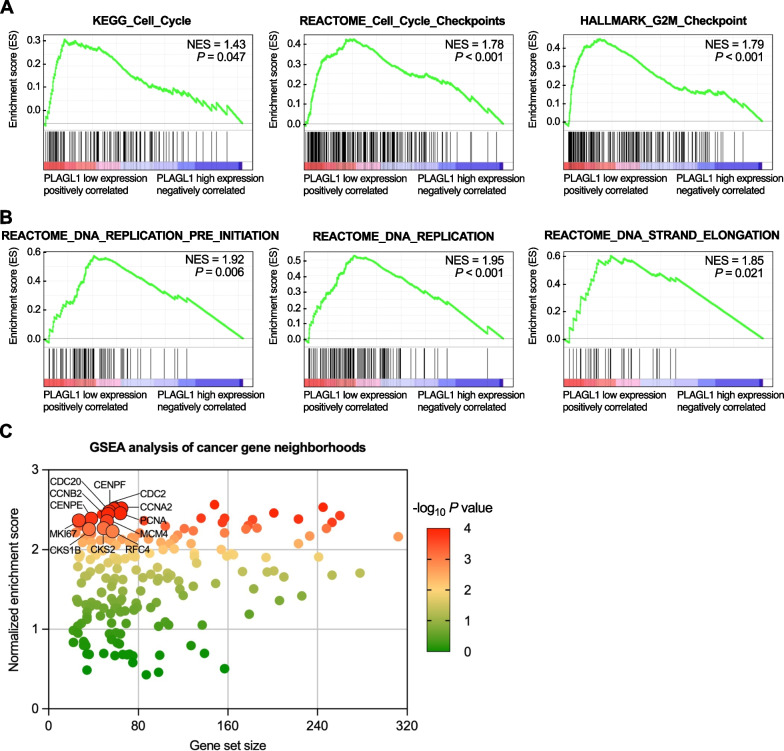
GSEA of *PLAGL1*-related gene sets from PAAD gene microarray data. **a**, **b** Cell proliferation- and DNA replication-associated pathways were enriched in PAAD tissues and negatively correlated with *PLAGL1* expression. **c** Gene set enrichment analysis for 380 cancer gene neighborhoods and *PLAGL1* expression in PAAD tissues

To determine the potential biological roles and significant pathways of *PLAGL1* in PAAD, we conducted GSEA based on high and low *PLAGL1* expression in the TCGA-PAAD cohort. The results indicated that multiple cell proliferation associated pathways were enriched in the lower *PLAGL1* expressing PAAD group, such as the cell cycle, cell cycle checkpoints, G2/M checkpoint, DNA replication preinitiation, DNA replication and DNA strand elongation pathways (Fig. 3a, b). Moreover, GSEA of cancer gene neighborhoods, defined by the expression domains of 380 cancer-related genes, suggested that *PLAGL1* was associated with cell cycle and cell proliferation operation related genes, which had significant *P* values and high normalized enrichment scores (Fig. 3c). Thus, we inferred that *PLAGL1* might inhibit the cell proliferation of PAAD cells by regulating the cell cycle and DNA replication.

### Integrated analysis reveals the prognostic value of *PLAGL1* in PAAD patients

**Fig. 4:**
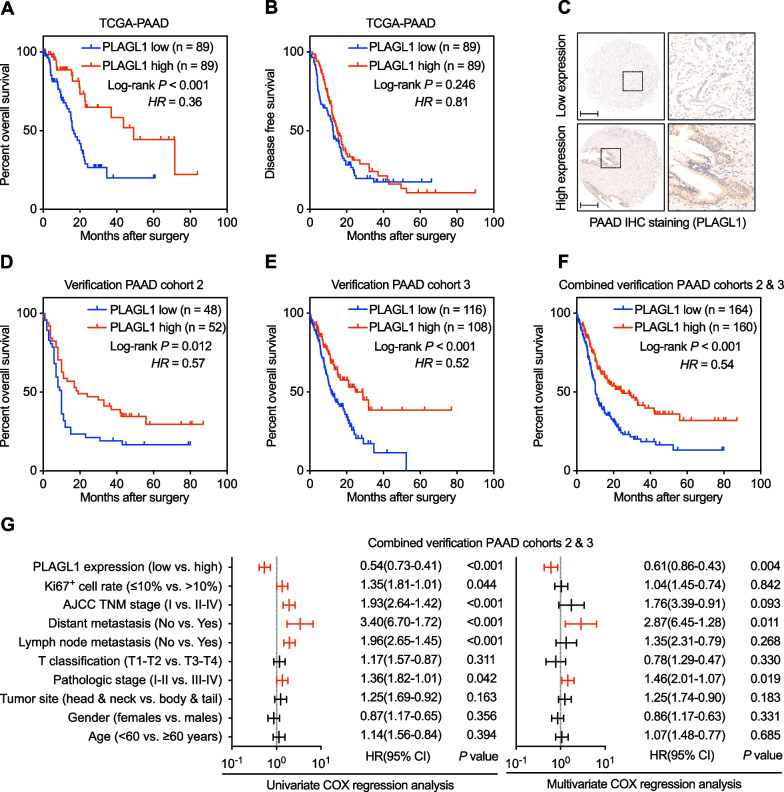
Prognostic prediction of *PLAGL1* in PAAD patients. **a**, **b** Log-rank test for comparing OS and DFS curves between *PLAGL1* mRNA high-expression and low-expression groups in TCGA-PAAD cohort. **c** Representative IHC staining of PLAGL1 protein high/low expression in our combined PAAD cohorts 2 and 3. **d**–**f** Log-rank test for comparing OS curves between PLAGL1 protein high-expression and low-expression groups analyzed by IHC staining of our PAAD verification cohort 2 and cohort 3. **g** Univariate and multivariate Cox regression analyses to investigate the prognostic value of PLAGL1 protein expression and PAAD clinicopathological parameters in our combined verification PAAD cohort 2 and 3. Scale bar = 200 μm

Overall survival (OS) assessment was performed to study whether PLAGL1 could be considered a new prognostic factor for PAAD patients. We found that a reduced *PLAGL1* level was clearly negatively associated with a worse OS of TCGA-PAAD cohort patients, while the association between *PLAGL1* and disease-free survival (DFS) was not significant (Fig. 4a, b). The analysis performed in our PAAD verification cohort 2 and 3 confirmed this association as well (Fig. 4c–f). Moreover, a univariate Cox regression model of cohorts 2 combined with cohort 3 was performed to predict the prognostic factors of PAAD. As shown in Fig. 4g, the protein level of PLAGL1, Ki67^+^ cell rate, AJCC TNM stage, distant metastasis, lymph node metastasis and pathologic stage showed prognostic value of PAAD patients in predicting their overall survival (Fig. 4g). Among them, PLAGL1 expression, distant metastasis and pathologic stage were independent factors of outcome according to multivariate Cox regression of PAAD patients in combined cohorts 2 and 3. Thus, these analyses suggested that, PLAGL1 might have value as a new prognostic factor for PAAD patients’ overall survival.

### The impact of PLAGL1 in preventing PAAD cell proliferation in vitro

**Fig. 5:**
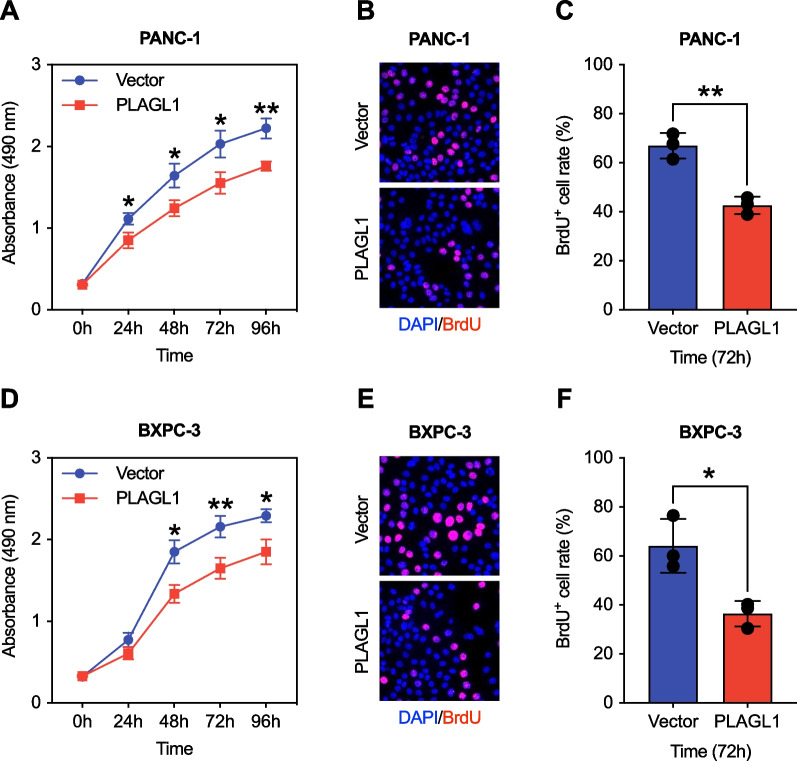
PLAGL1 overexpression decressed pancreatic cancer cell proliferation in vitro. **a** MTS analysis of PANC-1 transfected with *PLAGL1* overexpression vector or control vector. **b**–**c** Relative cell proliferation of PANC-1 transfected with *PLAGL1* overexpression vector or control vector by BrdU assay. **d** MTS analysis of BXPC-3 transfected with *PLAGL1* overexpression vector or control vector. **e**–**f** Relative cell proliferation of BXPC-3 transfected with *PLAGL1* overexpression vector or control vector by BrdU assay. ***P < 0.001; **P < 0.01; *P < 0.05

To study the potential impact of PLAGL1 on the proliferation function of pancreatic cancer cells, MTS and BrdU incorporation assays were performed to determine pancreatic cancer cell proliferation rates. Compared with the group transfected with control vector, PANC-1 cells with PLAGL1 overexpression showed decreased proliferation rates (Fig. 5a–c). The same effects were confirmed in BXPC-3 cells with PLAGL1 overexpression as well (Fig. 5d–f). Therefore, our data indicated that PLAGL1 had the impact of preventing the proliferation of pancreatic cancer cells.

## Discussion

Multiple studies have confirmed that *PLAG* family genes act as transcription factors of nuclear proteins or cofactors of other proteins in the malignant progression of multiple tumors. *PLAG1*, located on chromosome 8q12, is mostly mutated in pleomorphic adenoma of the salivary gland. It was originally recognized as a tumor suppressor gene that regulates the cell cycle and apoptosis [17]. However, recent studies gave a different opinion. The activation of *PLAG1* might be the critical event in the development of multiple human cancers; for example, upregulation of *PLAG1* was found in anoikis-resistant lung cancer cell lines and was required for lung metastasis [18]. *PLAG1* was also reported as a key transcription factor in driving the triple negative breast cancer phenotype [19]. *PLAGL2*, located on chromosome 20q11.21, is closely homologous with *PLAG1* and includes the indistinguishable consensus binding sequence GRGGC(N)6-8RGGK [4]. Overexpression of both *PLAG1* and *PLAGL2* has been verified to mediate the activation of *IGF-II* in NIH3T3 cells and human 293 kidney cell lines [4]. Since *IGF-II* plays a vital role in mitogenesis during embryonic development and carcinogenesis [20, 21], it can be supposed that *PLAG1* and *PLAGL2* can also work as proto-oncogenes regulating cell mitogenesis in multiple cancers. In fact, *PLAGL2* was primarily discovered to work as a transcription factor in the pathogenesis of acute myeloid leukemia [22, 23]. *PLAGL2* was recently reported to promote hepatocellular carcinoma progression, metastasis and erlotinib tolerance via the EGFR signaling pathway [24]. Wu et al. [25] also found that *PLAGL2* could activate USP37 expression and stabilize Snail1 to promote the proliferation and migration of gastric cancer cells. *PLAGL1*, located on chromosome 6q24, was primarily observed to function as a tumor suppressor gene in multiple cancers, similar to *p53* [26]. It has been primarily confirmed as a tumor suppressor gene in multiple cancers, such as large B-cell lymphoma and lung, gastric, colorectal, breast, ovarian and prostate cancer [6–10, 27]. However, much evidence suggests that *PLAGL1* might work as an oncogenic factor in many brain tumors [13, 14]. As a result, the role of *PLAGL1* in tumors is not yet clearly understood. Moreover, all three *PLAG* family genes have rarely been reported in PAAD.

In this study, we found that the *PLAGL1* expression level was significantly decreased in PAAD tissues in the TCGA database and GEO database and then verified this result in our three independent PAAD cohorts. Clinicopathological analysis based on the TCGA-PAAD cohort and our PAAD verification cohorts revealed that *PLAGL1* was negatively correlated with the Ki67^+^ cell rate and pathologic stage, indicating that *PLAGL1* might suppress the growth of PAAD. Therefore, we performed GSEA based on high and low *PLAGL1* expression in the TCGA-PAAD cohort and identified that *PLAGL1* expression was negatively correlated with the cell cycle and DNA replication pathways. As reported previously, PLAGL1 can bind the GC-rich motif of proximal promoter regions in imprinted genes and genes regulating extracellular matrix composition, and induce physiological cell cycle exit with contact inhibition. Such function shares with the biological role of p53 in different contexts [28]. In addition, GSEA of cancer gene neighborhoods also suggested that multiple genes in the gene set correlated with *PLAGL1* could regulate cell proliferation as well. For example, CDC20 could promote exit from mitosis and inhibit cell proliferation [29]; CENPF could promote cell mitosis resulting in enhanced tumor growth [30]; and CKS1B was confirmed to contribute to the ubiquitination and proteasome degradation of p27^Kip1^, inducing cell proliferation and inhibiting cell apoptosis [31]. The capacities of these *PLAGL1* nearby genes somehow imply the similar function of *PLAGL1*. According to MTS and BrdU assays, we found a decreased proliferation rate of pancreatic cancer cells with PLAGL1 overexpression, consistent with above deductions. Furthermore, we demonstrated that the reduced mRNA expression and protein expression of *PLAGL1* were both inversely correlated with a worse OS of PAAD patients, indicating a new potential marker for predicting PAAD patient prognosis.

Currently, PAAD is still one of the most life-threatening digestive malignancies worldwide. Although much focus and effort have been put into the study of PAAD, ranging from molecular mechanisms to surgical and drug therapies, the effect of the treatment has gained no clear improvement during the last two decades. Its 5 years survival rate is still less than 10% [2]. However, since neoadjuvant therapy was recommended to all borderline resectable PAAD patients in the 2016 NCCN clinical practice guidelines of PAAD (http://www.nccn.org/), approximately 30% of borderline resectable PAAD patients regained opportunities for R0 resection, resulting in longer OS. To obtain a radical operation, the tumor in patients who underwent neoadjuvant therapy should be inhibited from growth and, even better, shrunk. Thus, further understanding of the detailed mechanisms of PAAD proliferation and growth are particularly urgent. This study revealed the function of *PLAGL1* in reducing PAAD progression and its previously unrecognized prognostic value, providing new insight into the tumor biology of PAAD and providing us with a novel prognostic marker and therapeutic strategy that warrant investigation.

## Conclusion

In conclusion, the present study detected the differential expression of *PLAG* family genes in PAAD patients and suggested that downregulated *PLAGL1* might act as a biomarker in predicting poor prognosis and one of important factors in increasing cell proliferation in PAAD. Its expression level was significantly correlated with the Ki67^+^ cell rate, pathologic stage and overall survival in PAAD. The results of this study provide us with a novel prognostic marker and therapeutic strategy for PAAD, which deserves further study.

## Supplementary Information

**Additional file 1: Supplemental Table 1.** Clinical characteristics of 382 PAAD patients in our 3 independent verification cohorts.

**Additional file 2: Supplemental Table 2.** Sequences of qPCR primers

**Additional file 3: Supplemental Table 3.** Correlation analysis between clinical characteristics and PLAGL1 expression in our PAAD verification cohort 2.

**Additional file 4: Supplemental Table 4.** Correlation analysis between clinical characteristics and PLAGL1 expression in our PAAD verification cohort 3.

**Additional file 5: Supplemental Table 5.** Correlation analysis between clinical characteristics and PLAGL1 expression in our PAAD verification cohort 2 combined 3.

## Data Availability

The datasets supporting conclusions of this article are available in TCGA (https://portal.gdc.cancer.gov) and GEO (www.ncbi.nlm.nih.gov/geo/), respectively. The data used in the current study are available from the corresponding author upon reasonable request.

## Abbreviations

PAAD: Pancreatic adenocarcinoma
PLAG: Pleomorphic adenoma gene
TCGA: The Cancer Genome Atlas
GEO: Gene Expression Omnibus
qPCR: Quantitative polymerase chain reaction
IHC: Immunohistochemistry
ZFP: Zinc finger proteins
PLAGL1: Pleomorphic adenoma gene-like 1
PLAGL2: Pleomorphic adenoma gene-like 2
GSEA: Gene set enrichment analysis
FFPE: Formalin-fixed paraffin-embedded
OS: Overall survival
DFS: Disease free survival
SD: Standard deviation
BrdU: Bromodeoxyuridine
