# Supplementary material for: PLAGL1 is associated with prognosis and cell proliferation in pancreatic adenocarcinoma
Source: BMC Gastroenterol. 2023 Jan 4;23:2. doi: 10.1186/s12876-022-02609-y (PMC9811725; doi:10.1186/s12876-022-02609-y)
Supplement: Supplementary file 1 — Additional file 1: Supplemental Table 1. Clinical characteristics of 382 PAAD patients in our 3 independent verification cohorts. [file 12876_2022_2609_MOESM1_ESM.docx]

**Supplemental** Table 1. Clinical characteristics of 382 PAAD patients in our 3 independent verification cohorts.

| **Characteristics** | **Verification samples (n = 382)** | | |
| --- | --- | --- | --- |
|  | **Cohort 1 (n = 58)** | **Cohort 2 (n = 100)** | **Cohort 3 (n = 224)** |
| **Follow-up time (OS)** | | |  |
| Months, Median (ranges) | N.A | 10.0 (0.0-87.0) | 10.1 (0.1-77.0) |
| **Age** | | |  |
| Years, mean ± SD | 64.8±7.2 | 61.7±11.3 | 66.9±9.9 |
| <60 years, n (%) | 18(31.0) | 47(47.0) | 54(24.1) |
| ≥60 years, n (%) | 40(69.0) | 53(53.0) | 170(75.9) |
| **Gender** | | |  |
| Female, n (%) | 24(41.4) | 37(37.0) | 95(42.4) |
| Male, n (%) | 34(58.6) | 63(63.0) | 129(57.6) |
| **Tumor location** | | |  |
| Head/neck, n (%) | 42(72.4) | 60(65.9) | 153(68.3) |
| Body/tail, n (%) | 16(27.6) | 31(34.1) | 71(31.7) |
| **Pathologic stage** | | |  |
| Ⅰ-Ⅱ, n (%) | 39(67.2) | 69(69.0) | 140(62.5) |
| Ⅲ-Ⅳ, n (%) | 19(32.8) | 31(31.0) | 84(37.5) |
| **T classification** | | |  |
| T1-T2, n (%) | 45(77.6) | 78(79.6) | 118(52.7) |
| T3-T4, n (%) | 13(22.4) | 20(20.4) | 106(47.3) |
| **Lymph node metastasis** | | |  |
| No, n (%) | 32(55.2) | 54(58.1) | 153(68.3) |
| Yes, n (%) | 26(44.8) | 39(41.9) | 71(31.7) |
| **Distant metastasis** | | |  |
| No, n (%) | 56(96.6) | 98(98.0) | 214(95.5) |
| Yes, n (%) | 2(3.4) | 2(2.0) | 10(4.5) |
| **AJCC TNM stage** | | |  |
| Ⅰ, n (%) | 23(39.7) | 40(42.1) | 85(37.9) |
| Ⅱ-Ⅳ, n (%) | 35(60.3) | 55(57.9) | 139(62.1) |
| **Ki67^+^ cell rate** | | |  |
| ≤10%, n (%) | N.A | 64(64.0) | 144(64.3) |
| >10%, n (%) | N.A | 36(36.0) | 80(35.7) |
| **PLAGL1 expression** | | |  |
| Low, n (%) | 28(50.0) | 48(48.0) | 116(51.8) |
| High, n (%) | 28(50.0) | 52(52.0) | 108(48.2) |

PAAD, pancreatic adenocarcinoma; OS, overall survival; N.A, not applicable; SD, standard deviation.
