# Supplementary material for: PLAGL1 is associated with prognosis and cell proliferation in pancreatic adenocarcinoma
Source: BMC Gastroenterol. 2023 Jan 4;23:2. doi: 10.1186/s12876-022-02609-y (PMC9811725; doi:10.1186/s12876-022-02609-y)
Supplement: Supplementary file 2 — Additional file 2: Supplemental Table 2. Sequences of qPCR primers [file 12876_2022_2609_MOESM2_ESM.docx]

**Supplemental** Table 2. Sequences of qPCR primers

| **Name** |  | **Primer sequence (5'-3')** |
| --- | --- | --- |
| *PLAG1* | Forward | CAAAATGGGAAGGATTGGATT |
|  | Reverse | TCAGATGATCTTTCCGGTGA |
| *PLAGL1* | Forward | ACCCATTCTCCCCAGAAATC |
|  | Reverse | TGCCTCTTATAGCCCAGCAT |
| *PLAGL2* | Forward | ACCCACTCAGCCCAGAAAC |
|  | Reverse | GGTAGCCCAGCTTCGTATTG |
| *MKI67* | Forward | CTGCTTGTTTGGAAGGGGTA |
|  | Reverse | AGCCGTACAGGCTCATCAAT |
| *GAPDH* | Forward | TGCACCACCAACTGCTTAGC |
|  | Reverse | GGCATGGACTGTGGTCATGAG |
