# Supplementary material for: PLAGL1 is associated with prognosis and cell proliferation in pancreatic adenocarcinoma
Source: BMC Gastroenterol. 2023 Jan 4;23:2. doi: 10.1186/s12876-022-02609-y (PMC9811725; doi:10.1186/s12876-022-02609-y)
Supplement: Supplementary file 3 — Additional file 3: Supplemental Table 3. Correlation analysis between clinical characteristics and PLAGL1 expression in our PAAD verification cohort 2. [file 12876_2022_2609_MOESM3_ESM.docx]

**Supplemental** Table 3. Correlation analysis between clinical characteristics and PLAGL1 expression in our PAAD **verification** cohort 2.

| **Characteristics** | PLAGL1 **expression (n = 100)** | | ***OR*(95%*CI*)** | ***P* value** |
| --- | --- | --- | --- | --- |
|  | **Low (n = 48)** | **High (n = 52)** |  |  |
| **Age** | | |  |  |
| <60 years, n (%) | 19(39.6) | 28(53.8) | 1.78(0.80-3.94) | 0.153 |
| ≥60 years, n (%) | 29(60.4) | 24(46.2) |  |  |
| **Gender** | | |  |  |
| Female, n (%) | 15(31.3) | 22(42.3) | 1.61(0.71-3.67) | 0.253 |
| Male, n (%) | 33(68.8) | 30(57.7) |  |  |
| **Tumor location** | | |  |  |
| Head/neck, n (%) | 30(62.5) | 30(57.7) | 0.82(0.37-1.83) | 0.624 |
| Body/tail, n (%) | 18(37.5) | 22(42.3) |  |  |
| **Pathologic stage** | | |  |  |
| Ⅰ-Ⅱ, n (%) | 32(66.7) | 37(71.2) | 1.23(0.53-2.88) | 0.628 |
| Ⅲ-Ⅳ, n (%) | 16(33.3) | 15(28.8) |  |  |
| **T classification** | | |  |  |
| T1-T2, n (%) | 32(66.7) | 46(88.5) | 3.83(1.35-10.86) | 0.009 |
| T3-T4, n (%) | 16(33.3) | 6(11.5) |  |  |
| **Lymph node metastasis** | | |  |  |
| No, n (%) | 22(45.8) | 32(61.5) | 1.89(0.85-4.19) | 0.115 |
| Yes, n (%) | 26(54.2) | 20(38.5) |  |  |
| **Distant metastasis** | | |  |  |
| No, n (%) | 46(95.8) | 52(100) | / | 0.137 |
| Yes, n (%) | 2(4.2) | 0(0) |  |  |
| **AJCC TNM stage** | | |  |  |
| Ⅰ, n (%) | 11(22.9) | 29(55.8) | 4.24(1.78-10.10) | 8.7×10^-4^ |
| Ⅱ-Ⅳ, n (%) | 37(77.1) | 23(44.2) |  |  |
| **Ki67^+^ cell rate** | | |  |  |
| ≤10%, n (%) | 23(47.9) | 41(78.8) | 4.05(1.69-9.71) | 0.001 |
| >10%, n (%) | 25(52.1) | 11(21.2) |  |  |

PC, pancreatic cancer; OR, odds ratio; CI, confidence interval.
