# Supplementary material for: PLAGL1 is associated with prognosis and cell proliferation in pancreatic adenocarcinoma
Source: BMC Gastroenterol. 2023 Jan 4;23:2. doi: 10.1186/s12876-022-02609-y (PMC9811725; doi:10.1186/s12876-022-02609-y)
Supplement: Supplementary file 4 — Additional file 4: Supplemental Table 4. Correlation analysis between clinical characteristics and PLAGL1 expression in our PAAD verification cohort 3. [file 12876_2022_2609_MOESM4_ESM.docx]

**Supplemental** Table 4. Correlation analysis between clinical characteristics and PLAGL1 expression in our PAAD **verification** cohort 3.

| **Characteristics** | **PLAGL1 expression (n = 224)** | | **OR(95%CI)** | ***P* value** |
| --- | --- | --- | --- | --- |
|  | **Low (n = 116)** | **High (n = 108)** |  |  |
| **Age** | | |  |  |
| <60 years, n (%) | 25(21.6) | 29(26.9) | 1.34(0.72-2.47) | 0.354 |
| ≥60 years, n (%) | 91(78.4) | 79(73.1) |  |  |
| **Gender** | | |  |  |
| Female, n (%) | 53(45.7) | 42(38.9) | 0.76(0.44-1.29) | 0.303 |
| Male, n (%) | 63(54.3) | 66(61.1) |  |  |
| **Tumor location** | | |  |  |
| Head/neck, n (%) | 80(69.0) | 73(67.6) | 0.94(0.53-1.65) | 0.825 |
| Body/tail, n (%) | 36(31.0) | 35(32.4) |  |  |
| **Pathologic stage** | | |  |  |
| Ⅰ-Ⅱ, n (%) | 65(56.0) | 75(69.4) | 1.78(1.03-3.09) | 0.038 |
| Ⅲ-Ⅳ, n (%) | 51(44.0) | 33(30.6) |  |  |
| **T classification** | | |  |  |
| T1-T2, n (%) | 46(39.7) | 72(66.7) | 3.04(1.76-5.26) | 5.2×10^-5^ |
| T3-T4, n (%) | 70(60.3) | 36(33.3) |  |  |
| **Lymph node metastasis** | | |  |  |
| No, n (%) | 76(65.5) | 77(71.3) | 1.31(0.74-2.30) | 0.353 |
| Yes, n (%) | 40(34.5) | 31(28.7) |  |  |
| **Distant metastasis** | | |  |  |
| No, n (%) | 109(94.0) | 105(97.2) | 2.25(0.57-8.92) | 0.238 |
| Yes, n (%) | 7(6.0) | 3(2.8) |  |  |
| **AJCC TNM stage** | | |  |  |
| Ⅰ, n (%) | 30(25.9) | 55(50.9) | 2.97(1.70-5.21) | 1.1×10^-4^ |
| Ⅱ-Ⅳ, n (%) | 86(74.1) | 53(49.1) |  |  |
| **Ki67^+^ cell rate** | | |  |  |
| ≤10%, n (%) | 63(54.3) | 81(75.0) | 2.52(1.43-4.46) | 0.001 |
| >10%, n (%) | 53(45.7) | 27(25.0) |  |  |

PC, pancreatic cancer; OR, odds ratio; CI, confidence interval.
