# Supplementary material for: PLAGL1 is associated with prognosis and cell proliferation in pancreatic adenocarcinoma
Source: BMC Gastroenterol. 2023 Jan 4;23:2. doi: 10.1186/s12876-022-02609-y (PMC9811725; doi:10.1186/s12876-022-02609-y)
Supplement: Supplementary file 5 — Additional file 5: Supplemental Table 5. Correlation analysis between clinical characteristics and PLAGL1 expression in our PAAD verification cohort 2 combined 3. [file 12876_2022_2609_MOESM5_ESM.docx]

**Supplemental** Table 5. Correlation analysis between clinical characteristics and PLAGL1 expression in our PAAD **verification** cohort 2 combined 3.

| **Characteristics** | PLAGL1**expression (n = 324)** | | ***OR*(95%*CI*)** | ***P* value** |
| --- | --- | --- | --- | --- |
|  | **Low (n = 164)** | **High (n = 160)** |  |  |
| **Age** | | |  |  |
| <60 years, n (%) | 44(26.8) | 57(35.6) | 1.51(0.94-2.42) | 0.087 |
| ≥60 years, n (%) | 120(73.2) | 103(64.4) |  |  |
| **Gender** | | |  |  |
| Female, n (%) | 68(41.5) | 64(40.0) | 0.94(0.60-1.47) | 0.789 |
| Male, n (%) | 96(58.5) | 96(60.0) |  |  |
| **Tumor location** | | |  |  |
| Head/neck, n (%) | 110(67.1) | 103(64.4) | 0.89(0.56-1.40) | 0.609 |
| Body/tail, n (%) | 54(32.9) | 57(35.6) |  |  |
| **Pathologic stage** | | |  |  |
| Ⅰ-Ⅱ, n (%) | 97(59.1) | 112(70.0) | 1.61(1.02-2.55) | 0.041 |
| Ⅲ-Ⅳ, n (%) | 67(40.9) | 48(30.0) |  |  |
| **T classification** | | |  |  |
| T1-T2, n (%) | 78(47.6) | 118(73.8) | 3.10(1.94-4.94) | 1.4×10^-4^ |
| T3-T4, n (%) | 86(52.4) | 42(26.3) |  |  |
| **Lymph node metastasis** | | |  |  |
| No, n (%) | 98(59.8) | 109(68.1) | 1.44(0.91-2.27) | 0.117 |
| Yes, n (%) | 66(40.2) | 51(31.9) |  |  |
| **Distant metastasis** | | |  |  |
| No, n (%) | 155(94.5) | 157(98.1) | 3.04(0.81-11.44) | 0.085 |
| Yes, n (%) | 9(5.5) | 3(1.9) |  |  |
| **AJCC TNM stage** | | |  |  |
| Ⅰ, n (%) | 41(25) | 84(52.5) | 3.32(2.07-5.31) | 3.7×10^-7^ |
| Ⅱ-Ⅳ, n (%) | 123(75) | 76(47.5) |  |  |
| **Ki67^+^ cell rate** | | |  |  |
| ≤10%, n (%) | 86(52.4) | 122(76.3) | 2.91(1.81-4.69) | 7.8×10^-6^ |
| >10%, n (%) | 78(47.6) | 38(23.8) |  |  |

PAAD, pancreatic adenocarcinoma; OR, odds ratio; CI, confidence interval.
